# Analysis of risk factors associated with suicidality in children and adolescents with fetal alcohol spectrum disorder in Western Australia

**DOI:** 10.1111/acer.70039

**Published:** 2025-03-19

**Authors:** Grace Kuen Yee Tan, Sophia G. Connor, Sunee Quinn, James Fitzpatrick, Isabelle Adams, Carmela F. Pestell

**Affiliations:** ^1^ School of Psychological Science University of Western Australia Perth Western Australia Australia; ^2^ School of Medicine University of Western Australia Perth Western Australia Australia; ^3^ University of Western Australia Perth Western Australia Australia; ^4^ School of Psychological Science and Patches Australia University of Western Australia Perth Western Australia Australia; ^5^ The Kids Research Institute Australia Perth Children's Hospital Nedlands Western Australia Australia

**Keywords:** child protection system involvement, FASD, fetal alcohol Spectrum disorder, justice system engagement, mental health, risk factors, suicidality, young people

## Abstract

**Background:**

Individuals with fetal alcohol spectrum disorder (FASD) are at an elevated suicide risk compared with those in the general population. This public health issue warrants further research attention to help inform the development of prevention and intervention efforts. Our study is the first to characterize suicidality (i.e., suicidal ideation/suicide attempt) and explore associated risk factors in young individuals with FASD within the Australian context.

**Methods:**

Retrospective file reviews from a diagnostic clinic in Western Australia obtained data on demographic variables and risk factors, including psychosocial stressors (i.e., child protection and justice system involvement, history of abuse/neglect) and comorbid diagnoses (i.e., attention‐deficit‐hyperactivity disorder (ADHD), attachment disorder, conduct disorder, anxiety disorder, depression, substance use disorder, and sleep disorder). Data on suicidality were collected via formal suicide risk assessments and source documentation.

**Results:**

One hundred and ninety‐five participants diagnosed with FASD were included in the study (*M*_age_ = 11.75 years, range = 5–21 years). Of these, 40 (21%) reported suicidality, with the youngest being 5 years old. There was a significant positive correlation between suicidality and age. A greater proportion of individuals with FASD who had been involved with the justice system (*n* = 30, 35%) reported suicidality. Participants with attachment disorder (*n* = 19, 34%), conduct disorder (*n* = 10, 40%), substance use disorder (*n* = 14, 50%), and depression (*n* = 12, 60%) had significantly higher rates of suicidality than individuals without these comorbidities. The risk of suicidality increased in participants with comorbid depression (OR = 4.20) after controlling for age as a covariate.

**Conclusion:**

These findings add to the growing body of evidence that highlights the vulnerability of individuals with FASD to suicidality compared with the general population, underscoring the need for targeted, culturally safe suicide intervention/prevention efforts.

## Graphical abstract

Individuals with FASD face a higher risk of suicide, highlighting the need for targeted prevention efforts. Our study analyzed suicidality in 195 Australian individuals with FASD aged 5–21. Suicidality was reported by 21%, with the youngest aged 5. Risk was higher in older individuals and those with justice involvement (35%), attachment disorder (34%), conduct disorder (40%), substance misuse (50%), and depression (60%). Depression increased suicide risk fourfold. Findings emphasize the importance of culturally led intervention strategies.

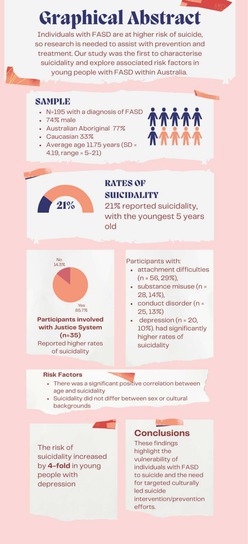

## INTRODUCTION

Fetal alcohol spectrum disorder (FASD) is a diagnostic term for a range of neurodevelopmental impairments resulting from prenatal alcohol exposure (PAE). Individuals with FASD often experience negative outcomes that develop over time due to a complex interaction between FASD symptoms, comorbid conditions, and psychosocial factors (Pei et al., 2011). A seminal study highlighted that 94% of individuals living with FASD experience at least one mental health condition, such as depression, in their lifetime (Streissguth et al., 1996). Individuals with FASD are also vulnerable to early life adversity associated with adverse childhood experiences (ACEs), making them vulnerable to mental health problems (Felitti et al., 2019). A growing body of research shows that those with FASD are also at an increased suicide risk across their lifespan.

The term suicidality encompasses suicidal ideation and suicide attempts, and data indicate that suicidality rates in individuals with FASD are much higher across all age groups compared with the general population (3%–9%), with estimated lifetime rates up to 19% in children, 39% in adolescents, and 55% in adults with FASD (Flannigan, Wrath, et al., 2022b). Additionally, research with American adolescents living with FASD found that 35% had experienced suicidal ideation in the last 12 months (O'Connor et al., 2019), and a FASD Canadian study reported 26% had experienced suicidal ideation/attempts, with 35% of those aged 13–24 years experiencing suicidality (Flannigan, McMorris, et al., 2022a). In a Swedish study of adoptees with FASD aged 18–28 years from Eastern Europe, 88% had been diagnosed with a psychiatric condition, and 21% had attempted suicide (Landgren et al., 2019). While suicidality is a broader societal concern, the higher rates among young people with FASD highlight the need for an increased understanding of the associated characteristics and risk factors to reduce unnecessary deaths. However, this issue has been limited by a lack of research, particularly within Australia. One study explored behavioral problems in children (*N* = 105) in remote Western Australia (WA), 21 of whom were living with FASD, and reported that of the four children who talked about killing themselves, three had been diagnosed with FASD (Tsang et al., 2017). While this study was limited in sample size, it indicates that further research on suicidality in young people with FASD with a more representative sample is warranted.

A range of biopsychosocial factors may contribute to suicide risk in individuals with FASD (Flannigan, McMorris, et al., 2022a). Those with lived experience of FASD often encounter emotional regulation difficulties, contributing to co‐occurring mental health conditions (Flannigan, McMorris, et al., 2022a; Temple et al., 2019) and impairments in executive functioning, such as disinhibition (Rasmussen et al., 2008). Poor impulse control has been associated with suicidal behavior in young people without FASD (McHugh et al., 2019) as well as those with other types of neurodevelopmental conditions, such as attention‐deficit‐hyperactivity disorder (ADHD) and autism (Gagliano et al., 2024). Additionally, high rates of adverse childhood experiences (ACEs) are documented in young people with FASD (Flannigan et al., 2021; Kambeitz et al., 2019; Tan et al., 2022), with research suggesting that those with four or more ACEs in the general community are at an elevated risk of health‐risk behavior, such as suicide (Felitti et al., 2019).

Other risk factors of suicidality, such as justice system involvement and substance use challenges, have also been identified in youth with FASD (Flannigan, McMorris, et al., 2022a). Additionally, children with child protection system involvement have three times the odds of suicide compared to those living with their biological parents (Palmer et al., 2021). However, much less is known about the relationship between child protection involvement and suicidality in young people with FASD.

Within Australia, the rates of FASD in the general population are not currently known, in part due to poor sector awareness and diagnostic constraints (McLean, 2022). Concerns have also been raised about the rate of FASD in some Aboriginal communities, with an Australian study finding that the prevalence of FASD was reported to be between 12% and 19% in a remote community (Fitzpatrick et al., 2015, 2017). However, it is important to acknowledge that the prevalence rate of FASD will differ across different Indigenous communities due to varying levels of awareness, attitudes, and relationships with alcohol (McLean, 2022). Notably, both social and cultural determinants of health (e.g., socioeconomic disadvantage, structural racism, and intergenerational trauma) must be considered when trying to understand the intersection between FASD and suicide in these communities (Dudgeon et al., 2017; Gonzales et al., 2021). Decolonizing disability for Australian Aboriginal and Torres Strait Islander communities requires adequate resourcing of the Aboriginal Community Controlled Health Sector (Hollinsworth, 2013; Puszka et al., 2022) and addressing the deeply rooted structural inequities (e.g., systemic racism and socioeconomic marginalization) that often hinder access to timely interventions/diagnostic services, education, and culturally sensitive care, all of which are pertinent to promoting more positive outcomes (Blagg et al., 2020).

Enhancing our knowledge in this area is critical to direct FASD and suicide prevention efforts and to inform the development of evidence‐based interventions, particularly for younger cohorts that may be amenable to early intervention (Burns et al., 2021; Deren, 2019). Therefore, this study aimed to characterize suicidality and explore the associated risk factors in a sample of West Australian young people with FASD.

## MATERIALS AND METHODS

### Sampling procedures

The current study employed a retrospective cohort design of 480 individuals who attended a multidisciplinary clinic in Perth, WA, between 2016 and 2019, whereby 226 participants met Australian FASD diagnostic criteria (Bower & Elliott, 2016). FASD referrals for the diagnostic clinic came from government agencies, health practitioners, and schools. A pediatrician and a neuropsychologist were always part of the FASD diagnostic team, while the involvement of other health professionals (e.g., speech pathologist, occupational therapist) occurred on a case‐by‐case basis. Suicidality was not reported in participants below age 5, so 16 participants aged 2–4 were excluded. Given our study's focus on young people, we also removed 15 individuals who were over 21 using the median absolute method, resulting in a sample size of 195 participants with FASD.

### Sample characteristics

Most individuals were male (*n* = 142, 73%) and identified as Australian Aboriginal peoples (*n* = 151, 77%). The mean age was 11.75 years (SD = 4.19, range = 5–21); 136 participants (70%) had been involved with the child protection system. The age of criminal responsibility in WA is 10 years old (Urbas, 2000). For individuals aged 10 and above (*n* = 133), 85 (64%) were involved with the justice system at the time of the assessment. More than half of the total sample (*n* = 110, 56%) had a recorded history of abuse/neglect.

### Data collection

All participants (or legal guardians) provided consent at the time of assessment for their data to be used for this research. All FASD diagnostic reports and source documents (e.g., medical reports) were retrospectively reviewed by the first author (in consultation with the last author) to ensure consistency in data collected. Clinicians routinely gathered information on participants' demographics and psychosocial stressors (e.g., history of abuse/neglect, child protection and justice system involvement, illicit substance use) through clinical interviews and source documents (e.g., allied health reports and government records). Individuals with justice system involvement had been charged with offenses and were on remand.

Information on participants' psychiatric/neurodevelopmental diagnoses was collected from medical and allied health reports. While all participants were evaluated for additional comorbidities during the assessment, these were only diagnosed by team clinicians if the diagnostic criteria according to the Diagnostic and Statistical Manual of Mental Disorders, Fifth Edition (DSM‐5) were met (American Psychiatric Association, 2013). Specifically, these were attention‐deficit hyperactivity disorder (ADHD), attachment disorder, conduct disorder, anxiety disorder, depression, substance use disorder, and sleep disorder.

Due to the broad nature of the term “suicidality,” this study focused on past suicide ideation (expressing suicidal ideas in the past month) and history of past suicide attempts. Clinicians also evaluated the client's suicide risk at the time of the diagnostic assessment. Interview questions on suicidality were from a Brief Risk Assessment interview framework (see Supplementary Material). While the suicide risk assessment was comprehensive, only responses to these questions (i.e., “In the last month, have you felt so bad/low that you thought about killing yourself? Have you attempted suicide in the past month?”) were captured in the dataset. Specifically, participants who endorsed either one of these questions were coded as “Yes” for suicidality. This aligned with the definition of suicidality as a broad term that encompasses suicidal behavior, such as ideation and attempts (Carballo et al., 2020).

### Ethics and partnership with relevant stakeholders

Ethics approval was obtained from the WA Aboriginal Health Ethics Committee (WAAHEC) (HREC Approval Number: 901), and an Aboriginal community reference group, with lived experience of FASD, was formed before the commencement of the project. The community reference group members were approved by WAAHEC after extensive consultation and with approval from local Aboriginal organizations. It was led by IA, an Australian Aboriginal and Torres Strait Island woman who lives in WA and is an author on this paper. To ensure the research protocols were culturally led, members of the reference group were involved in identifying research aims, interpreting the findings, and drafting and reviewing the manuscript. Regular confidential yarning circles were arranged and led by IA to facilitate a culturally safe place to discuss the methodology, key findings, and interpretations of the study findings.

### Data analysis

All statistical analyses were conducted using IBM SPSS‐22. The Benjamini–Hochberg procedure was used to correct for multiple comparisons (Benjamini & Hochberg, 2000). Characteristics of the sample and the rates of suicidality were explored using descriptive statistics. A point‐biserial correlation examined the relationship between age (continuous variable) and suicidality (Yes/No). A series of chi‐squared analyses were employed to investigate group differences in suicidality (Yes/No) based on demographic factors (i.e., sex and cultural background), psychosocial stressors (i.e., child protection involvement, justice system involvement, and history of abuse/neglect) and comorbid conditions (i.e., ADHD, attachment disorder, conduct disorder, anxiety disorder, depression, substance use dependence, and sleep disorder). Results from these chi‐squared analyses and past literature on the risk factors of suicidality in young people with FASD (Flannigan et al., 2021; Flannigan, McMorris, et al., 2022a; Kambeitz et al., 2019; Tan et al., 2022; Temple et al., 2019) were used to determine the predictors entered in the multivariate logistic regression described below. A post hoc descriptive analysis was conducted to explore the frequency and percentage of participants with a history of abuse/neglect who also had been involved with child protection services.

A multivariate logistic regression was conducted with age entered at Block 1 as the confounding variable. At Block 2, predictor variables, including justice system involvement, conduct disorder, attachment disorder, depression, and substance use disorder, were entered simultaneously. Assumptions for the logistic regression, such as linearity and independence of errors, were tested and met.

## RESULTS

### Demographic factors and suicidality

Of the total sample (*N* = 195), 40 participants (21%) reported suicidality (i.e., suicidal ideation/suicide attempts). The mean age of this subgroup was 14.38 (SD = 4.24). The youngest participant who reported suicidality was 5 years old. There was a significant positive correlation between age and suicidality, *r*_pb_ (195) = 0.32, *p* < 0.001. In contrast, suicidality did not differ between sexes or cultural backgrounds (Table 1).

**TABLE 1:** Rates of suicidality and demographic factors.

Demographic factors	Suicidality/*n* (%)	*X* ^2^	Unadjusted *p*‐value	Hochberg threshold	Effect size/*φ*
Sex					
Male (*n* = 142)	31 (22)	0.56	0.456	0.042	0.053
Female (*n* = 53)	9 (17)				
Cultural background^a^					
Caucasian (*n* = 44)	9 (21)	0.001	0.991	0.050	0.001
Australian Aboriginal peoples (*n* = 151)	31 (21)				

^a^ No other ethnicities represented.

### Psychosocial stressors and suicidality

Chi‐squared analysis shows that a greater proportion of individuals with FASD who were justice‐involved (*n* = 30, 35%) reported suicidal ideation/suicide attempts than those without (*n* = 5, 10%). However, there were no differences in the rates of suicidality based on child protection involvement and a history of abuse/neglect (Table 2). A post hoc descriptive test indicates that 83% (*n* = 92) of participants with a history of abuse/neglect had also been involved with child protection services.

**TABLE 2:** Rate of suicidality and psychosocial stressors.

Psychosocial stressors	Suicidality/*n* (%)	*X* ^2^	Unadjusted *p*‐value	Hochberg threshold	Effect size/*φ*
Child protection involvement					
Yes (*n* = 136)	23 (17)	3.58	0.059	0.025	0.135
No (*n* = 59)	17 (29)				
Justice system involvement**					
Yes (*n* = 85)^a^	30 (35)	9.79	0.002	0.013	0.322
No (*n* = 48)	5 (10)				
History of abuse/neglect					
Yes (*n* = 110)	23 (21)	0.24	0.876	0.046	0.011
No (*n* = 85)	17 (20)				

^a^ The total *n* for justice system involvement was 133, as only those aged 10 and above were considered in the analysis.

**p < 0.01.

### Comorbid diagnoses and suicidality

A comorbid diagnosis of ADHD was present in almost half of the cohort (*n* = 87, 45%). Less frequent comorbid conditions were sleep disorder (*n* = 63, 32%), attachment disorder (*n* = 56, 29%), anxiety disorder (*n* = 48, 25%), substance use disorder (*n* = 28, 14%), conduct disorder (*n* = 25, 13%), and depression (*n* = 20, 10%). Chi‐squared analyses indicated that participants with attachment disorder, conduct disorder, substance use disorder, and depression had significantly higher rates of suicidality than individuals without these comorbidities (Table 3). These relationships also remained statistically significant after corrections for multiple comparisons were applied. In contrast, suicidality did not differ between participants with comorbid conditions of ADHD, anxiety disorder, and sleep disorder.

**TABLE 3:** Rate of suicidality and comorbid conditions.

Comorbid conditions	Suicidality/*n* (%)	*X* ^2^	Unadjusted *p*‐value	Hochberg threshold	Effect size/*φ*
ADHD					
Yes (*n* = 87)	23 (26)	3.38	0.066	0.029	0.132
No (*n* = 108)	17 (16)				
Sleep disorder					
Yes (*n* = 63)	17 (27)	2.39	0.122	0.033	0.111
No (*n* = 132)	23 (17)				
Attachment disorder**					
Yes (*n* = 56)	19 (34)	8.67	0.003	0.017	0.211
No (*n* = 139)	21 (15)				
Anxiety disorder					
Yes (*n* = 48)	13 (27)	1.69	0.194	0.038	0.093
No (*n* = 147)	27 (18)				
Substance use disorder***					
Yes (*n* = 28)	14 (50)	17.44	<0.001	0.008	0.299
No (*n* = 167)	26 (16)				
Conduct disorder*					
Yes (*n* = 25)	10 (40)	6.68	0.010	0.021	0.185
No (*n* = 170)	30 (18)				
Depression***					
Yes (*n* = 20)	12 (60)	21.31	<0.001	0.004	0.331
No (*n* = 175)	28 (16)				

*p < 0.05, **p < 0.01, ***p < 0.001.

### Risk factors of suicidality

After controlling for age, the logistic regression shows that only comorbid depression remained a statistically significant predictor of suicidality. Specifically, the risk of suicidality increased by fourfold in young people with depression compared to those without this comorbid diagnosis (Table 4). Justice system involvement, substance use disorder, conduct disorder, and attachment disorder were not significant predictors of suicidality after accounting for age as a covariate in the regression model.

**TABLE 4:** Multivariate logistic regression predicting suicidality in young people with fetal alcohol spectrum disorder (FASD).

	Nagelkerke *R* square	Cox and Snell *R* square	*B*	SE	Wald	*p*‐Value	Odds ratio (95% CI)
Block 1	0.10	0.15					
Age			0.28	0.08	12.47	<0.001	1.32 (1.13, 1.53)
Block 2	0.20	0.29					
Age			0.16	0.09	3.04	0.081	1.18 (0.98, 1.41)
Justice system involvement^a^			0.51	0.61	0.69	0.405	1.65 (0.50, 5.50)
Attachment disorder			0.50	0.48	1.09	0.296	1.65 (0.64, 4.22)
Substance use disorder			0.62	0.53	1.40	0.237	1.86 (0.66, 5.23)
Conduct disorder			0.78	0.56	1.95	0.163	2.18 (0.73, 6.50)
Depression*			1.39	0.56	6.12	0.013	4.02 (1.34, 12.11)

^a^ Only participants aged 10 and above were included.

* *p* < 0.05.

## DISCUSSION

This study provides preliminary evidence indicating high rates of suicidality in individuals aged 5–21 years with FASD and informs associated risk factors. Suicidality was associated with increasing age as well as justice involvement. Participants with attachment disorder, conduct disorder, substance use dependence, and depression had significantly higher rates of suicidality than individuals without these comorbidities. Notably, the risk of suicidality increased by fourfold in participants with comorbid depression after controlling for age as a confounding factor.

These results offer a unique West Australian perspective, which is pertinent given that the National FASD Strategic Action Plan aims to reduce the associated impact of FASD in Australia (Australian Department of Health, 2018). The rate of suicidality in this study (21%) aligns with that in similarly aged individuals with FASD in other countries. For example, 19% (suicide threats) for children 6–11 years in America (Streissguth et al., 1996), 17%–27% (suicide attempts/ideation) in children/youth in Canada (Burns et al., 2021; Flannigan, Wrath, et al., 2022b); and 21% (self‐harm/suicide attempts) of justice‐involved youth aged 13–18 years also reported in Canada (Deren, 2019). The suicidality rates in this study (21%) were also higher than the 12‐month prevalence of suicidal thoughts and behaviors in young people in the general Australian population (4.9%) (Australian Bureau of Statistics, 2022). The high rate of suicidality in individuals with FASD highlights their vulnerability, which may be partly attributable to a complex array of systemic barriers, including a lack of culturally safe diagnostic services, poor sector awareness, and stigma related to diagnosis and service engagement (Panton et al., 2023).

Additionally, the nature of the neuropsychological impairments that are associated with PAE could contribute to suicidality. That is, impulsive–aggressive traits, impulsive decision making, deficits in inhibitory control, impaired decision making, and hopelessness are associated with an increased risk for suicidality in young people, regardless of any underlying diagnoses (Wasserman et al., 2021). Individuals with FASD can struggle with self‐regulation, leading to intense emotional arousal and greater externalizing behaviors, such as self‐harm and suicide attempts (Temple et al., 2019). Therefore, interventions that target emotional and behavioral regulation may hold promise in reducing suicidality in individuals with FASD.

Furthermore, the influence of demographic factors, psychosocial stressors, and comorbidities may also contribute to the higher suicidality in this vulnerable population. Unfortunately, the rates of FASD in the Australian general population are currently unknown (McLean, 2022). This has implications for resource allocation for FASD‐related interventions and prevention efforts, as it is challenging to gauge the scale of FASD across different communities. Additionally, policymakers lack the evidence to prioritize FASD as a public health issue to enable equitable resource distribution. It is also noteworthy that there is a lack of FASD diagnostic services and evidence‐based interventions for individuals with FASD to reduce risks and vulnerabilities across the lifespan (Reid et al., 2020), as well as a lack of knowledge and confidence among Australian clinicians in conducting FASD assessments, contributing to underdiagnosis and missed opportunities for early intervention (Brown et al., 2017). There is a need to advocate for improved FASD awareness and training in the Australian health sector, particularly in relation to suicide interventions. Until adequate resources are applied, it is not possible to properly contextualize adversity and trauma for individuals affected by FASD. Positively, the Australian Government's recent response to the Senate Community Affairs References Committee report acknowledges the importance of improving national FASD prevalence data and access to FASD support services, aligning with the objectives in the National Fetal Alcohol Spectrum Disorder (FASD) Strategic Action Plan 2018–2028.

### Suicidality and demographic characteristics

The onset of suicidality usually begins during the pubertal phase, with increased susceptibility to negative social cues and risk‐taking behavior continuing throughout adolescence (Hawton et al., 2012). Our findings are in keeping with this trend, as participants' suicide risk increased as they matured. It is possible that challenges, such as substance misuse, incarceration, and housing/employment instability, increase during the teenage years, driving individuals to adopt maladaptive coping mechanisms or attempt suicide (McLachlan et al., 2020). Additionally, current evidence indicates there is typically a lack of appropriate resources and services for individuals with FASD, particularly support for transitioned‐age youth between the ages of 16 and 25, which, coupled with reduced coping skills, likely increases their vulnerability to suicidality (Brown et al., 2019). A notable finding was that participants as young as 5 years old reported suicidality in our study, consistent with research suggesting that the conceptualization of death can occur in children as early as preschool (Slaughter, 2005). While the possibility of suicidal ideation in very young children is well recognized, there is a paucity of Australian research on this important public health issue. One study did find that rates of suicidal ideation and suicidal behavior were 19.1% and 3.5%, respectively, in a depression treatment‐seeking (*n* = 288) and healthy control sample (*n* = 26) of 3‐ to 6.11‐year‐old American children (Luby et al., 2019). It has also been reported that less than half of children who die by suicide have received mental health care (Luby et al., 2019). This highlights an urgent need for early interventions in child suicide prevention, especially considering individuals with FASD are already vulnerable to a host of early life adversity. Early screening of PAE and FASD symptoms during infant health evaluations would be ideal. Screening at least on first contact with child protection or mental health services would also be beneficial, given it has been recommended that very young children who have been exposed to violent events and/or are presenting with symptoms of depression and irritability should undergo a suicide risk assessment as part of any standard clinical interview (Luby et al., 2019).

The rates of suicidality in this study did not differ based on sex, unlike the general population, where it has been shown that females more often attempt suicide and males have higher suicide mortality partly due to their suicide method (Fasher et al., 1997). In FASD research, mixed findings on this issue have been reported, with some results indicating no sex‐related differences in suicide (Flannigan, Wrath, et al., 2022b) and others indicating higher rates of serious suicide attempts (29%) in males (O'Connor et al., 2019), in contrast to higher rates of serious suicidal ideation (41%) and suicide attempts (35%) in females with FASD (Peled & Smith, 2014). As our study did not differentiate between specific suicidal behaviors (i.e., ideation, suicide attempts) or mechanisms of suicide, this lack of sex‐based differentiation may hide subtle differences in suicide‐related behaviors between sexes. The present study was also skewed due to a high number of male participants. Future studies exploring suicidal behaviors in young people with FASD, focusing on sex‐based differences, would help clarify this discrepancy.

Cultural background was not associated with higher suicidality in this study, which aligns with some earlier studies exploring suicidal ideation between Aboriginal and non‐Aboriginal Australians (Sawyer et al., 2010). However, our results are inconsistent with more recent work, which documented higher rates of suicide, self‐harm, and suicidal ideation in Aboriginal groups in Australia (Dickson et al., 2019) and higher rates of suicidality among First Nation Canadian children with FASD compared to those without FASD (Brownell et al., 2019). The results of this study may be attributable to the underreporting of suicidality due to stigma associated with suicidal ideation/attempts or a lack of disclosure secondary to possible poor rapport with the clinician, which can inhibit help‐seeking (Bowden et al., 2020). Furthermore, it is possible that the Western conceptualizations of suicidal behavior may not apply to Australian Aboriginal populations (Tighe et al., 2015), leading to culturally relevant questions being inadvertently omitted from the risk assessment interview. For example, members of the Aboriginal community advisory group in this study reported that knowing or witnessing someone close who lost their life to suicide was highlighted as a significant risk for suicide for Aboriginal peoples. Alternatively, resiliency and protective factors, such as connection to country, culture, spirituality, and empowerment in relation to cultural/community well‐being within this FASD cohort, may account for the findings (Dudgeon et al., 2017), and are consequently worthy of further exploration and intervention efforts. Additionally, the “cultural continuity” model suggests that community‐level cultural factors protect against suicide in Australian Aboriginal youth by increasing connectedness with their past and future cultural lineage (Gibson et al., 2021).

### Suicidality and psychosocial stressors

Children with FASD represent an already vulnerable population as they often come from disadvantaged backgrounds (i.e., low socioeconomic status, educational disadvantage, and family instability) and have a high rate of ACEs (Flannigan et al., 2021; Kambeitz et al., 2019; Tan et al., 2022) without access to early diagnosis and intervention (Blagg et al., 2020). Notably, a large population‐based study indicates that child‐reported family conflict is a known risk factor for suicidality (Janiri et al., 2020), and individuals with four or more ACEs in the general population are at an elevated suicide risk (Felitti et al., 2019). A history of maltreatment has also been associated with a threefold increase in suicidality in individuals with FASD across their lifespans (Flannigan, McMorris, et al., 2022a), with associated repercussions of grief and loss due to early mortality, potentially magnifying transgenerational trauma within families. However, a history of abuse/neglect was not associated with suicidality in our study. Post hoc analysis indicates that a substantial proportion (84%) of participants with a history of abuse/neglect had also been involved with child protection. The low suicidality rate in this subgroup was unexpected, given research indicating that children with a child protection services history are less likely to access mental health services and are more vulnerable to suicidality (Duong et al., 2021). This finding may be partly due to child protection services having better identification of suicidality in young people compared with other Australian government services, presumably leading to early access to appropriate supports (O'Hare et al., 2023). Nonetheless, the ongoing significant gaps in the healthcare needs of children in care continue to be recognized, with more investment needed in intensive family support programs (SNAICC, 2023), particularly given that Western Australia has the highest overrepresentation of Aboriginal and Torres Strait Islander children in out‐of‐home care in Australia (SNAICC, 2023). Future research mapping required services and evaluating the effectiveness of culturally led mental health interventions in this vulnerable cohort will be important.

This study also found higher rates of suicidality in young people with FASD who were justice‐involved when age was not accounted for as a covariate, aligning with previous research that focuses on the vulnerability of these individuals to suicidality (Sawyer et al., 2010). Individuals involved in the justice system, regardless of FASD diagnosis, are at significant risk for suicidal ideation, attempts, or completion, with suicide, for example, accounting for more than a third of deaths of juveniles in confinement (Scott et al., 2015). This is due to a myriad of reasons, including a higher frequency of comorbid disorders, such as depression (Wasserman et al., 2010) and neurodevelopmental impairments, as well as prior suicide attempts and substance misuse (Scott et al., 2015). Members of the Aboriginal advisory group of this study highlighted that the enduring impacts of colonization, systemic racism, and intergenerational trauma have contributed to the high rates of suicidality in Aboriginal young people with FASD in the justice system. They added that the difficulties of living with undiagnosed or unsupported FASD in the prison system, together with the cycles of incarceration, stigma, and cultural disconnection, can contribute to feelings of hopelessness and despair, leading to high suicide rates. These issues call for policies that address the broader systemic inequities and strategies that prioritize prevention efforts, early diagnosis, and support for individuals with FASD (Hamilton et al., 2021; Hayes et al., 2014; Hewlett et al., 2023). Surprisingly, this study found that justice system involvement was not a significant risk factor for suicidality in the presence of age as a significant confounding variable, possibly due to access to psychological services. As previously mentioned, there is a need for interventions and support services aimed at older individuals with FASD, regardless of their justice involvement.

### Suicidality and comorbidities

Suicidality rates were higher in participants with comorbid attachment disorder, substance use, depression, or conduct disorder.

#### Attachment disorder

The finding that attachment disorder is associated with suicidality is consistent with past literature demonstrating a relationship between insecure attachment and suicidality (Sheftall et al., 2014). Depending on the type of attachment disorder, these individuals may be less likely to share feelings and experience more difficulties with forming meaningful relationships with others (Miranda et al., 2019). Protective factors against suicidality include parent and child interactions with good support, which may not necessarily be possible for individuals with a history of disrupted attachment (Ati et al., 2021). These issues are likely heightened in individuals with FASD, many of whom experience complex trauma leading to child protection or justice system involvement, which would prevent regular contact with family (Tan et al., 2022). Interventions that focus on improving attachment with caregivers could potentially mitigate suicide risk in children and adolescents with FASD. Furthermore, community‐based interventions, such as the Parent‐Child Assistance Program (PCAP) to support mothers who struggle with substance misuse during pregnancy, may also help to improve mental health outcomes and interrupt intergenerational involvement with child protection (Rasmussen et al., 2012; Symons et al., 2022). Additionally, well‐established frameworks, such as Joiner's Interpersonal‐Psychological Theory of Suicide approach, may be relevant to the assessment, intervention, and prevention of suicidal risk for children and adolescents with FASD (Stewart et al., 2017).

#### Substance use disorder

Individuals with FASD are known to experience substance use problems at over five times the rate of the general population (Streissguth et al., 1996), with higher rates of suicidality (Flannigan, McMorris, et al., 2022a). Substance misuse is a known risk factor for suicide (Pompili et al., 2012), and unsurprisingly, it remains so in the FASD population (Flannigan, McMorris, et al., 2022a). In our study, young people with FASD and substance abuse were 2.8 times more likely to report suicidality than those without substance abuse. The underlying reasons for a higher propensity for substance misuse in individuals with FASD likely include a complex interplay of biological vulnerability and environmental and epigenetic factors (Flannigan et al., 2020). Illicit substance use also exacerbates psychological distress and inhibits adaptive coping strategies, contributing to suicidal behaviors. Substance misuse may also result in impaired judgment, mood changes, and impulsivity (already an issue for individuals with FASD) and can distance individuals from their peers who do not endorse such activities (Pompili et al., 2012). Furthermore, substance dependence is common in individuals experiencing stressful circumstances, such as poor academic performance, legal problems, or interpersonal conflict, all frequent occurrences within the FASD population (Pompili et al., 2012). Hence, interventions that target substance misuse in young people with FASD and assist them with stressful psychosocial circumstances in a culturally safe manner could reduce the burden of suicidality across their entire lifespan.

#### Depression

Notably, having a comorbid diagnosis of depression increased the risk of suicidality by 4‐fold in this clinical population after considering age as a covariate. However, other factors, such as justice system involvement, substance misuse, conduct, and attachment problems, were not significant predictors of suicidality after controlling for age. The importance of depression as a risk factor aligns with past research, particularly for youths (Beautrais, 2000). For example, the presence of comorbid psychopathology (i.e., affective disorders, disruptive/conduct disorders, and substance dependence) is associated with a significantly higher risk of suicide death among youth in the general community (Fleischmann et al., 2005; Gili et al., 2019; Soole et al., 2015). The increasing prevalence of mental health disorders during adolescence is considered an explanation for why suicide risk tends to increase with age (Soole et al., 2015). Compared with other FASD research, the rate of suicidality in individuals with depression in our study (60%) was higher than that found in other studies (37% and 38%; Flannigan, Wrath, et al., 2022b; O'Connor et al., 2019), possibly due to the higher number of participants in the present study from vulnerable backgrounds involving contact with child protection and justice. Nonetheless, emotional regulation difficulties (Temple et al., 2019) and mood disturbance (Flannigan, Wrath, et al., 2022b) experienced by individuals with FASD increase the likelihood of suicidality in this clinical population. Our findings are also in keeping with a recent narrative review pointing to depression as a significant underlying risk factor for suicidality in neurodevelopmental conditions, leading the authors (Gagliano et al., 2024) to put forward a model in which emotional dysregulation and ACEs directly lead to suicidality by interacting with symptoms of depression. This proposed theoretical model (Gagliano et al., 2024) is worthy of further exploration, particularly as it may inform more targeted prevention and intervention approaches. Currently, there is a lack of evidence‐based interventions focusing on emotional regulation difficulties and treating mental health problems, such as depression in individuals with FASD (Flannigan et al., 2020). Our results suggest that screening and monitoring of depression are particularly important given the role that these symptoms appear to play in predicting suicidality in young people with FASD. Our findings also suggest that in terms of clinical management, clinicians should always consider formulating a safety plan for those with comorbid depression, given the increased risk (fourfold) of suicidality in this clinical population.

### Strengths, limitations and future research directions

The strengths of this study include the consistent manner in which the psychosocial stressors and comorbid diagnoses were documented by clinicians, allowing for an accurate comparison between individuals. This study also employed a systematic approach to capturing suicidality in young people with FASD, although future research could better characterize suicidal ideation and types of suicide attempts. Importantly, Australian Aboriginal people who formed the reference group were consulted to ensure that the research methods and interpretations of findings were culturally appropriate. Limitations included a lack of detailed information collected regarding the nature and type of justice system and/or child protection involvement, as well as missing data on current or past interventions addressing suicidality and other protective factors. There was also no context regarding suicidality in terms of severity, frequency, and chronicity. Due to the unique sociodemographic composition of our clinic sample (i.e., child protection and/or justice background), our findings may not be representative of the Australian FASD population as a whole; however, they may be transferable to similar disadvantaged settings.

Notably, the use of a mainstream suicide risk assessment measure in Aboriginal participants was highlighted as a major limitation by members of the Aboriginal advisory group in this study. While all clinicians involved in the risk assessments had completed cultural awareness training to enable culturally safe practices, such as decolonization and trauma‐informed yarning about Aboriginal Australia, the suicide measure employed in this study did not explore suicide risk (e.g., witnessing or knowing someone close who suicided) and protective factors (e.g., cultural resiliency) specific to Aboriginal peoples. The members of the Aboriginal advisory group highlighted that the exploration of culturally specific suicide risk and protective factors represents a critical area of future research, with findings to inform targeted prevention efforts and intervention strategies specific to Aboriginal peoples. It would be important for future researchers to use a codesign approach to facilitate the inclusion of a wider representation of Aboriginal experiences and voices (Williams et al., 2024). Positively, members of the advisory group acknowledged that the questions included in the suicide risk measure in this study were practical and realistic, which they considered appropriate for use in a yarning format with Aboriginal young people with FASD despite the limitations outlined above. During consultancies with members of the Aboriginal advisory group, it was also emphasized that the development of a suicide risk assessment tool for Aboriginal young people with FASD should include a visual modality in the response options to address any issues with standard Australian English and challenges with expressive language that could prevent meaningful participation in the suicide risk assessment.

Nonetheless, these results have important implications and provide direction for future studies. The absence of a control group precludes us from attributing the high rates of suicidality to PAE alone and represents a critical area for future research. Future studies must overcome the methodological limitations discussed above and consider the varied nature of suicidality to establish more robust prevalence estimates. Ideally, a greater exploration into the individual's experience of suicide (e.g., frequency, chronicity, and severity) with associated risk factors could occur, particularly in vulnerable groups, such as justice‐involved youth. The impact of overall comorbidity on suicide risk is also worth exploring in this cohort. For example, it has been established that emotional distress, substance misuse, disruptive behavior, and high fear/anger significantly increase suicidal behavior in adolescents (Nock et al., 2013). Our study did not explore the specific relationship between alcohol use and suicidality. However, this association is important given current statistics indicating that alcohol is the most commonly used substance among young people in Australia, and the average age of alcohol use initiation (16 years old) is younger than illicit substance use (20 years old) (Australian Institute of Health and Welfare, 2020).

Exploring the protective factors for suicidality and strengths in individuals with FASD would be valuable to help build resilience and self‐esteem. For example, this could include focusing on participants' access to interventions, medications, a stable home environment and a strong support network. While questions, such as “What is stopping you from killing yourself?”, were part of the Brief Risk Assessment used in this study, this protective factor was not captured in our database and is also worthy of exploration. Importantly, considerations should be given to cultural strengths and how these can be incorporated into suicide prevention and intervention programs to maximize treatment gains and engagement in First Nations communities.

## CONCLUSIONS

This study provides novel evidence of the rate of suicidality in a large sample of young West Australian people with FASD. Overall, results highlight the vulnerability of individuals with FASD to suicidality from multiple perspectives, including age, psychosocial stressors, and comorbid conditions. Consequently, tailored suicide prevention efforts that consider the compounding layers of risks associated with FASD are essential to promote better outcomes in young people with FASD. Furthermore, our findings highlight a significant need for culturally led interventions and access to evidence‐based therapies/support that address emotional regulation difficulties, substance misuse problems, challenges with attachment, and symptoms of depression to prevent and reduce suicidality in young people with FASD.

## AUTHOR CONTRIBUTIONS

Conceptualization: GKY Tan and CF Pestell. Data Curation: GKY Tan, SG Connor, CF Pestell, and JP Fitzpatrick. Formal Analysis: GKY Tan and CF Pestell. Investigation: GKY Tan, SG Connor, and CF Pestell. Methodology: GKY Tan, I Adams, and CF Pestell. Resources: JP Fitzpatrick. Writing Initial Draft: GKY Tan, SG Connor, S Quinn, and CF Pestell. Writing—review and editing: CF Pestell, GKY Tan, SG Connor, S Quinn, I Adams, and JP Fitzpatrick.

## CONFLICT OF INTEREST STATEMENT

Dr J. Fitzpatrick is Director of PATCHES Australia. S.G. Connor is related to C.F. Pestell. No other conflicts to declare.

## Supporting information


Appendix S1

## Data Availability

The data that support the findings of this study are available on request from the corresponding author. The data are not publicly available due to privacy or ethical restrictions.
