# Supplementary material for: Analysis of risk factors associated with suicidality in children and adolescents with fetal alcohol spectrum disorder in Western Australia
Source: Alcohol Clin Exp Res (Hoboken). 2025 Mar 19;49(5):1149–60. doi: 10.1111/acer.70039 (PMC12098806; doi:10.1111/acer.70039)
Supplement: Supplementary file 1 — Appendix S1 [file ACER-49-1149-s001.docx]

**Supplementary Material**

Suicide Risk Assessment Interview Template

*“In the last month, have you felt so low/bad that you thought about killing yourself? If yes, how often do you think about killing yourself? Has anything happened recently that has caused you to have these thoughts? Have you thought about how you would do it? Do you have an active plan to try and take your life? Have you ever attempted suicide in the past month? Has anyone in your family attempted suicide? What is stopping you from killing yourself?”*
